# Emergency Department Use Among Recently Homeless Adults in a Nationally Representative Sample

**DOI:** 10.5811/westjem.59054

**Published:** 2023-08-11

**Authors:** Caitlin Ryus, Elina Stefanovics, Jack Tsai, Taeho Greg Rhee, Robert A. Rosenheck

**Affiliations:** * Department of Emergency Medicine, Yale University School of Medicine, New Haven, Connecticut; † U.S. Department of Veterans Affairs New England Mental Illness Research, Education, and Clinical Center, West Haven, Connecticut; ‡ Department of Psychiatry, Yale University School of Medicine, New Haven, Connecticut; § VA National Center on Homelessness among Veterans, Washington, DC; ∥ School of Public Health, University of Texas Health Science Center at Houston, Houston, Texas; ¶ Department of Public Health Sciences, University of Connecticut School of Medicine, Farmington, Connecticut

## Abstract

**Introduction:**

In this study we examined the association of homelessness and emergency department (ED) use, considering social, medical, and mental health factors associated with both homelessness and ED use. We hypothesized that social disadvantage alone could account for most of the association between ED use and homelessness.

**Methods:**

We used nationally representative data from the National Epidemiologic Survey on Alcohol and Related Conditions (NESARC-III). Emergency department use within the prior year was categorized into no use (27,674; 76.61%); moderate use (1–4 visits: 7,972; 22.1%); and high use (5 or more visits: 475; 1.32%). We used bivariate analyses followed by multivariable-adjusted logistic regression analyses to identify demographic, social, medical, and mental health characteristics associated with ED use.

**Results:**

Among 36,121 respondents, unadjusted logistic regression showed prior-year homelessness was strongly associated with moderate and high prior-year ED use (odds ratio [OR] 2.31 and 7.34, respectively, *P* < 0.001). After adjusting for sociodemographic factors, the associations of homelessness with moderate/high ED use diminished (adjusted OR [AOR] 1.27 and 1.62, respectively, both *P* < 0.05). Adjusting for medical/mental health variables, alone, similarly diminished the association between homelessness and moderate/high ED use (AOR 1.26, *P* < .05 and 2.07, *P* < 0.001, respectively). In a final stepwise model including social and health variables, homelessness was no longer significantly associated with moderate or high ED use in the prior year.

**Conclusion:**

After adjustment for social disadvantage and health problems, we found no statistically significant association between homelessness and ED use. The implications of our findings suggest that ED service delivery must address both health issues and social factors.

Population Health Research Capsule What do we already know about this issue?

*Previous research among patient populations in clinical settings demonstrated a strong association between homelessness and high frequency ED use*.

What was the research question?

*Do social disadvantage and health-related factors account for much of the extensive ED use by homeless adults?*

What was the major finding of the study?

*Adjusted for social and health factors, homelessness and ED use were not significantly associated (AOR 1.27, 95% CI 0.79–2.03)*.

How does this improve population health?

*The complex interplay between social and medical issues should encourage the development of service delivery models linking these intersecting dimensions of need*.

## INTRODUCTION

Emergency departments (EDs) have long served as a healthcare safety net for the medical needs of marginalized populations in the US, such as people experiencing homelessness.^1^ Over the past several years, there has been increasing recognition that in providing this service, EDs play a distinct role in delivering “social emergency medicine” to address the structural determinants of poor health such as poverty, racism, inadequate housing, and food insecurity.^2^^–^^4^ “Emergency department use is costly,^5^ and some question the appropriateness and efficiency of addressing social problems within the ED and healthcare system, especially in the US where social services are limited.^5^^,^^6^

Previous studies have shown that ED patients are far more likely to be homeless than other adults.^7^^,^^8^ A multisite study conducted in Northeastern Pennsylvania estimated that the prevalence of homelessness in EDs ranged from 7–18%.^7^ At one ED in New York City, 14% of patients were homeless and 25% had been concerned about becoming homeless during the prior two months.^8^ Homelessness is specifically associated with high levels of ED use.^9^^–^^14^ National data from the Veterans Health Administration found that patients experiencing homelessness were 6.6 times more likely than others to have more than 25 ED visits annually.^15^ Furthermore, patients experiencing homelessness are four times more likely than others to re-present to the ED within three days of a prior evaluation.^16^ Although homelessness is strongly associated with high ED use, it may not be independently associated with such use since many social and medical factors may drive this association; however, this needs to be empirically examined.

Previous research on ED utilization among patients experiencing homelessness has been almost exclusively based on data from patient populations sampled in clinical settings, potentially biasing our understanding of how homelessness relates to ED use in the general population.^7^^–^^11^^,^^13^^–^^16^ Few studies have examined ED use in nationally representative samples that included non-health service users. The few available reports from community-based studies among homeless individuals suggest that only a small minority (8–12%) use the ED more than three times per year.^17^^,^^18^ Thus, there is a need to examine this issue in a nationally representative sample.

In this study, we used a nationally representative survey, the National Epidemiologic Survey on Alcohol and Related Conditions (NESARC-III), to examine the association of recent homelessness and factors that may be associated with ED use.^19^ We hypothesized that social disadvantage (eg, poverty, racism, educational attainment, and neighborhood environment) might account for much of the extensive ED use by homeless adults, although we suspected that health-related factors would also play a role. We thus sought to examine evidence to clarify how social, medical, and mental health factors play into the association between homelessness and ED use among the most socially disadvantaged sectors of the US population.

## METHODS

### Data Source and Study Sample

We performed a cross-sectional analysis to assess the association between homelessness and ED use using data from NESARC-III. The NESARC-III is a nationally representative survey of 36,906 adults, which includes information on experiences of prior-year homelessness and emergency care utilization as well as demographic and recent social, medical, and mental health characteristics.^19^ This data allows examination of the association of both recent homelessness and other factors that are likely to be associated with ED use, thus offering an examination of the independent association of homelessness and ED use when social, medical, and mental health factors are taken into consideration. The survey was sponsored by the National Institute on Alcohol Abuse and Alcoholism (NIAAA) and conducted between April 2012–June 2013 among the non-institutionalized US civilian population ≥18 years old.^19^ Multistage probability sampling was used to randomly select persons from this population. Primary sampling units were individual counties or groups of contiguous counties. Secondary sampling units consisted of area segments of census-defined blocks. Households within the sampled secondary sampling units were then selected. Finally, eligible adults within the sampled households were randomly selected.^19^ An initial 43,364 eligible sample persons were identified, and 36,309 participated in the NESARC-III, while 7,055 were classified as nonresponders, for a person-level response rate of 84.0%.^19^

A total of 36,309 respondents completed the Alcohol Use Disorder and Associated Disabilities Interview Schedule, DSM-5 version (AUDADIS-5), a fully structured, computer-assisted diagnostic interview conducted by trained NIAAA interviewers.^20^ Institutionalized individuals (eg, in nursing homes, prisons, hospitals, or shelters) were excluded along with active duty military personnel. Racial/ethnic minorities were oversampled to assure representative analysis. Data was adjusted for oversampling and nonresponse and then weighted to represent the US civilian population based on the 2012 American Community Survey.^21^ Informed consent was electronically recorded, and respondents received $90 for participation. Institutional review boards (IRB) at the US National Institutes of Health and Westat, Inc. (Rockville, MD) approved the study protocol. This study was approved by the IRBs of the Department of Veterans Affairs Connecticut Healthcare System and Yale School of Medicine.

## MEASURES

### ED Utilization

We measured the primary outcome variable, ED utilization, based on self-report by respondents and categorized into a three-level variable representing no use (0 visits), moderate use (1–4 visits), and high use (5 or more visits) in the prior year.

### Sociodemographics

Sociodemographic characteristics included age, gender, race, marital status, annual household income, level of education, employment status, military service, rural vs urban residence, and health insurance coverage.

### Social History

Social history variables addressed homelessness, incarceration, interaction with law enforcement, parental social history, adverse childhood experiences such as sexual abuse or neglect, experiences of racial discrimination, social contacts, and social support. We created two dichotomous homelessness variables that identified adults with homelessness in the past year and homelessness prior to the most recent past year. Lifetime homelessness was assessed with this question: “Since you were 15, did you have a time that lasted at least one month when you had no regular place to live—like living on the street or in a car?” A separate question—“In the last 12 months, have you at any time been homeless?”—was the independent variable of central interest in this study. A previous study using NESARC-III data reported the lifetime and one-year prevalence of homeless to be 4.2% and 1.5%, respectively.^22^

Other social history variables included questions such as “During the last 12 months, did you have serious trouble with the police or law?” which was coded into a dichotomous variable for police involvement. Experiencing racial discrimination was a continuous variable assessed from six questions within AUDADIS-5 that have been shown to have good validity and reliability for measuring experiences of racial discrimination.^22^ The six discrimination questions ask about experienced racial discrimination in six contexts: obtaining healthcare/health insurance; receiving care; in public; obtaining a job; being called racist names; being hit/threatened with harm. We used a Likert scale to assess the frequency of experiences of discrimination in the past year: 0 = never; 1 = almost never; 2 = sometimes; 3 = fairly often; or 4 = very often.

Parental history included experiences of incarceration, psychiatric hospitalization, suicide attempt or completion, and substance use. The extent of social support was assessed using the Interpersonal Support Evaluation List^23^^,^^24^: perceived availability of others to share activities, talk about one’s problems and from whom to potentially receive material support. Social contacts were assessed through a series of questions regarding how many people the respondents had contact with in the previous two weeks, which were summed to create an index of social contacts. Veterans were identified as those who responded to the question “Have you ever served on active duty in the U.S. Armed Forces, Military Reserves, or National Guard?” with “Yes, in the past, but not now.”

### Medical, Mental Health, and Service Use History

Medical history variables included number of medical comorbidities, up to 18; presence of moderate to severe pain; number of injuries in the past year; cancer history; body mass index (BMI) > 40; and mental- and physical health-related quality of life. Respondents were asked whether or not they had each of 18 medical conditions (eg, arthritis, diabetes, and insomnia) in the past 12 months. Those who responded positively were further asked, ‘‘Did a doctor or health professional tell you that you had [a medical condition]?’’ Using these two questionnaire items for each medical condition, we created a measure of chronic conditions experienced in the past year. Quality of life was measured using the Short Form-12, version 2 (SF-12), a reliable and valid measure of health status commonly used in population surveys.^25^^,^^26^ The 12 questions can be scored into subscales to yield a mental component summary (MCS) score and a physical component summary (PCS) score as well as overall subjective health status. The number of injuries reported by respondents was assessed by the question, “During the last 12 months, how many injuries have you had that caused you to seek medical help or to cut down your usual activities for more than half a day?” This variable was measured as a continuous variable.

We assessed lifetime or past year presence of DSM-5 mental health diagnoses with the AUDADIS-5 and included the following: mood disorders (major depressive disorder, bipolar I disorder, dysthymia); anxiety disorders (generalized anxiety disorder, specific phobia, panic disorder); post-traumatic stress disorder (PTSD), and eating disorder. We used AUDADIS-5 scoring for all disorders except schizophrenia/psychosis, which was addressed with the following question, “Did a doctor or other health professional tell you that you had schizophrenia or a psychotic illness or episode?” Personality disorders included antisocial, borderline, and schizotypal. Lifetime and past year substance use disorders (SUD) included alcohol use disorder, as well as cannabis, cocaine, opiate, heroin, stimulant, and sedative use disorders (considered together as non-alcohol drug use disorders), and tobacco use disorder.

Multimorbidity was addressed with dichotomous variables indicating the following: the presence of only one psychiatric diagnosis and another indicating two or more such diagnoses; the presence of only one SUD diagnosis and another indicating two or more such diagnoses. An additional measure captured the presence of both psychiatric disorder and SUD (dual diagnosis).

### Data Analysis

We used a series of bivariate analyses to evaluate the association of each demographic, social, or medical and mental health characteristic with each level of ED usage. Because there was inflated statistical power given the large sample size, we selected variables for inclusion in subsequent multivariable analyses based on effect sizes rather than *P*-values. We identified risk ratios > 1.5 or < 0.7 as representing substantial and meaningful effects for dichotomous variables.^27^ For continuous variables we used Cohen d as an indicator of effect size, with d > 0.20 or <−0.20 indicating at least a small effect size.^28^

We then conducted a series of four logistic regression analyses conducted separately with different sets of independent variables, all including past year homelessness. The first logistic regression was unadjusted and included only past year homelessness as the independent variable. The second model was adjusted only for demographic and social variables meeting criteria for substantial bivariate effects and thus evaluated the concurrent role of social determinants of health. A third logistic model examined only co-occurring medical and mental health variables showing substantial association with ED use in bivariate analyses. We included variables regarding parental suicide, drug use, and psychiatric hospitalization in the third (health) model, whereas parental prison history was included in the second model of non-medical social risk factors. Finally, we entered all variables with meaningful effect sizes per the above criteria into a stepwise multinomial logistic regression analysis with forward selection to identify a parsimonious set of statistically significant factors that were independently associated with moderate and high ED use. Since all these variables had passed the effect size screens on bivariate analysis, we applied a conventional *P* < 0.05 level of statistical significance to these models.

We computed standardized regression coefficients to allow identification of variables most strongly associated with ED use. Comparison of −2 log likelihood indicators were used to assess goodness of fit with larger values indicating superior fit. We performed all analyses using SAS version 9.4 (SAS Institute Inc, Cary, NC).

## RESULTS

### Sample

Of the total 36,121 respondents with complete data, 27,674 (76.61%) reported no ED use in the past year, 7,972 (22.07%) reported moderate ED use, and 475 (1.32%) reported high use. Having experienced homelessness within the past year was reported by 559 (1.55%) respondents, and 1,541 (4.27%) responded that they had experienced homelessness within their lifetime.

### Bivariate Correlates of ED Use

Bivariate analyses showed past year homelessness (relative risk [RR] = 6.83) to be among the three variables most strongly associated with high ED use, exceeded only by past year suicide attempt (RR = 11.51) and receipt of a diagnosis of schizophrenia or psychosis in the past year (RR = 8.61) (Tables 1–3). Demographic variables substantially associated with moderate and high ED use included receiving disability benefits (RR = 2.71 and RR = 7.68, respectively). Having a college education was protective (Table 1).

**Table 1.** Bivariate associations of demographic variables with emergency department use.

	ED use Group 1 N = 27,674	ED use Group 2 n = 7,972	ED use Group 3 n = 475	Bivariate analysis	
	0 visits	1–4 visits	≥5 visits	2 vs 1	3 vs 1
Variable	mean (SD)/%	mean (SD)/%	mean (SD)/%	RR/Cohen d^*^	RR/Cohen d^*^
Gender					
Male	49.15%	44.91%	36.57%	0.91	0.74
Age^*^	46.25 (17.48)	47.61 (18.76)	44.82 (17.59)	0.08	−0.08
Annual income					
<$20,000	20.80%	28.20%	51.05%	1.36	2.45
$20,000–40,000	23.47%	26.46%	25.81%	1.13	1.1
$40,000–60,000	22.51%	20.87%	11.77%	0.93	0.52
>$60,000	33.22%	24.47%	11.39%	0.74	0.34
Race					
Black	10.74%	15.01%	20.90%	1.4	1.95
White	66.26%	66.37%	61.79%	1	0.93
Hispanic	15.25%	13.17%	10.09%	0.86	0.66
Other	7.75%	5.45%	7.24%	0.7	0.93
Marital status					
Separated or divorced	12.81%	16.89%	25.09%	1.32	1.96
Widowed	5.27%	7.48%	8.54%	1.42	1.62
Never married	22.58%	21.82%	29.68%	0.97	1.31
Married or cohabitating	59.34%	53.81%	36.69%	0.91	0.62
Employment					
Receives disability	3.61%	9.77%	27.70%	2.71	7.68
Looking for work	6.98%	8.65%	13.99%	1.24	2
Other employment	16.92%	17.60%	18.89%	1.04	1.12
Retired	16.68%	20.76%	14.11%	1.24	0.85
Employed	72.36%	63.01%	49.85%	0.87	0.69
Military service					
Any military service	9.08%	11.94%	10.54%	1.32	1.16
Rurality					
Urban	78.96%	78.21%	75.06%	0.99	0.95
Highest level of education					
Pre-high school	12.17%	15.27%	23.65%	1.26	1.94
High school	25.01%	28.31%	31.24%	1.13	1.25
Pre-college	32.41%	35.29%	36.49%	1.09	1.13
College	30.42%	21.14%	8.62%	0.69	0.28
Health insurance coverage					
Medicaid	8.22%	16.58%	32.40%	2.02	3.94
VA Tricare	4.18%	6.42%	8.20%	1.53	1.96
Medicare	19.43%	27.71%	32.85%	1.43	1.69
Any insurance	79.43%	83.49%	85.30%	1.05	1.07
Private insurance	59.83%	51.99%	35.23%	0.87	0.59

Bivariate analyses compare moderate and high ED users to non-users.

* Denotes continuous variable with Cohen d for measure of association.

*ED*, emergency department; *RR*, relative risk; *VA*, Veterans Administration.

**Table 2.** Bivariate associations of social variables with emergency department use.

	ED use Group 1 n = 27,674	ED use Group 2 n = 7,972	ED use Group 3 n = 475	Bivariate analysis	
	0 visits	1–4 visits	≥5 visits	2 vs 1	3 vs 1
Variable	mean (SD)/%	mean (SD)/%	mean (SD)/%	RR/ Cohen d^*^	RR/ Cohen d^*^
Homelessness					
Past year	1.14%	2.59%	7.81%	2.27	6.83
Lifetime	3.39%	6.68%	14.84%	1.97	4.38
Incarceration history					
Police trouble in past year	1.30%	2.50%	5.41%	1.92	4.17
Incarcerated before age 18	3.33%	5.86%	12.47%	1.76	3.74
Incarcerated after age 18	9.56%	14.91%	24.47%	1.61	2.64
Social history and social support					
History of child neglect^*^	12.22 (5.02)	13.49 (6.21)	15.62 (8.35)	0.19	0.52
History of child sexual abuse^*^	4.37 (1.54)	4.76 (2.40)	5.49 (3.18)	0.17	0.47
Racial discrimination in the past year^*^	1.21 (.43)	1.29 (.52)	1.47 (.70)	0.14	0.48
Social support^*^	3.02 (.464)	2.96 (.51)	2.83 (.61)	−0.12	−0.37
Number of contacts in the past two weeks^*^	16.36 (15.08)	15.91 (15.08)	14.28 (14.28)	−0.02	−0.11
Parental history					
Parent with suicide attempt	2.81%	4.20%	10.46%	1.49	3.72
Parent with prison history	6.48%	11.49%	19.07%	1.77	2.94
Parent with suicide completion	0.86%	0.98%	2.42%	1.14	2.82
Parent with drug use history	4.99%	8.15%	11.26%	1.63	2.38
Parent psychiatric hospitalization history	4.74%	7.47%	11.26%	1.58	2.38

Bivariate analyses compare moderate and high ED users to non-users.

* Denotes continuous variable with Cohen’s d for measure of association.

*ED*, emergency department; *RR*, relative risk.

**Table 3.** Bivariate associations of medical and mental health variables with emergency department use.

	ED use Group 1 n = 27,674	ED use Group 2 n = 7,972	ED use Group 3 n = 475	Bivariate analysis	
	0 visits	1–4 visits	≥5 visits	2 vs 1	3 vs 1
Variable	mean (SD)/%	mean (SD)/%	mean (SD)/%	RR/Cohen d^*^	RR/Cohen d^*^
Psychiatric and substance use disorders					
Past year suicide attempt	0.10%	0.50%	1.16%	5.01	11.51
Schizotypal disorder	5.04%	10.10%	22.14%	2.01	4.4
Antisocial disorder	3.58%	6.48%	15.61%	1.81	4.37
Lifetime diagnosis of schizophrenia or psychosis	1.73%	3.57%	7.41%	2.07	4.29
Past year greater than one substance use disorder diagnosis	1.79%	3.54%	6.84%	1.98	3.82
Past year single drug use disorder diagnosis	3.15%	5.93%	11.30%	1.88	3.58
Past year greater than one psychiatric diagnosis	6.72%	13.51%	23.34%	2.01	3.47
Borderline personality disorder	7.96%	16.87%	29.72%	2.01	3.47
Past year dual diagnosis: psychiatric/ substance use disorder	4.00%	7.16%	9.48%	1.79	2.37
Past year single psychiatric diagnosis	12.57%	16.91%	24.93%	1.35	1.98
Multiple recurring traumas	12.86%	16.00%	22.00%	1.24	1.71
Lifetime alcohol use disorder diagnosis	27.97%	32.56%	38.13%	1.16	1.36
Past year single substance use disorder diagnosis	12.85%	15.03%	17.01%	1.17	1.32
Past year alcohol use disorder diagnosis	13.16%	16.05%	17.01%	1.22	1.29
Medical history					
Medical conditions (range 1–18)^*^	0.62 (0.97)	1.28 (1.48)	2.26 (1.97)	0.45	1.12
Number of injuries^*^	0.20 (2.11)	0.96 (4.50)	2.44 (8.00)	0.16	0.46
General health (scale of 1 to 5)^*^	2.33 (1.04)	2.84 (1.15)	3.64 (1.14)	1.11	0.46
Short Form-12 mental component^*^	51.62 (9.18)	48.63 (11.42)	43.37 (12.56)	−0.27	−0.75
Short Form-12 physical component^*^	50.91 (9.49)	45.51 (12.37)	36.68 (12.85)	−0.47	−1.23
Moderate or severe pain	15.80%	32.77%	63.90%	2.07	4.04
Any history of cancer	3.65%	6.20%	12.94%	1.70	3.55
BMI >40	3.76%	6.76%	10.78%	1.8	2.86

Bivariate analyses compare moderate and high users to non-users.

* Denotes continuous variable with Cohen’s d for measure of association.

*ED*, emergency department; *RR*, relative risk; *BMI*, body mass index.

Among the social variables (Table 2) associated with moderate and high ED use, homelessness in the past year (RR = 2.27 and RR = 6.83) and homelessness within one’s lifetime (RR = 1.97 and RR = 4.38) were associated with the highest relative risk. Experiencing trouble with the police (RR = 4.17) and history of incarceration before and after age 18 (RR = 3.74 and RR = 2.64) were also associated high ED use, as were adverse childhood events such as neglect, sexual abuse, parental suicide attempts, parental suicide completion, parental imprisonment, parental drug use, and parental psychiatric hospitalization. As Black race was associated with high ED use, it should be noted that experiencing racial discrimination in the past year was also significantly associated with high ED use.

Health status variables (Table 3) associated with moderate and high ED use included worse general health (Cohen d = 1.11 and 1.12), experiencing moderate or severe pain, and a BMI > 40. Higher scores on both the PCS and MCS were protective.

Among the mental health variables associated with ED use (Table 3), suicide attempt in the past year was the most strongly associated with both moderate (RR = 5.01) and high ED use (RR = 11.51). Moderate and high ED use were both associated strongly with personality disorders and diagnosis of schizophrenia or psychosis within one’s lifetime. Having more than one substance use disorder, more than one psychiatric disorder, or dual diagnosis within the past year were also all associated with moderate and high use (Table 3).

### Multivariate Multinomial Logistic Regression Analyses

**Figure 1.**
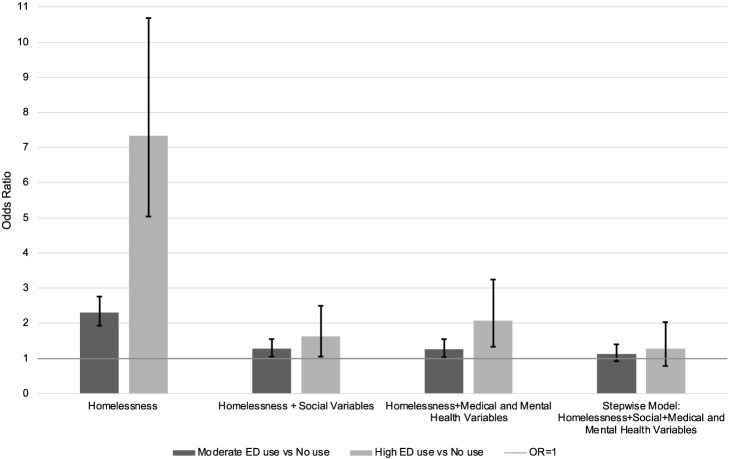
Association of past year homelessness with moderate and high ED use: Unadjusted and Adjusted Odds ratios from multinomial logistic regression models. *OR*, odds ratio.

Unadjusted logistic regression analysis demonstrated that people with experience of homelessness within the past year were approximately twice as likely to report moderate ED use (odds ratio [OR] 2.31; 95% confidence interval [CI] 1.93–2.76; *P* < 0.001) and seven times more likely to report high ED use (OR 7.34; 95% CI 5.04–10.68; *P* < 0.001) compared to those without past year experience (Figure 1).

After adjusting only for demographic, social variables, the association of homelessness and its statistical significance were greatly diminished as people with past year experience of homelessness were only 27% more likely to report moderate ED use than others (adjusted OR [AOR] 1.27; 95% CI 1.05–1.54; *P* = 0.014) and 62% more likely to report high ED use (AOR 1.62; 95% CI, 1.05–2.50; *P* = 0.030) (Figure 1).

The third model, which adjusted only for medical and mental health variables, also showed marked decline in ORs compared to the unadjusted model as participants experiencing past year homelessness were 26% more likely to report moderate use (AOR 1.26; 95% CI 1.03–1.55; *P* = 0.025) and about twice as likely as to have high ED use (AOR 2.07; 95% CI, 1.33–3.24; *P* = 0.001).

In the final stepwise model with forward selection at *P* < 0.05, including all substantially important variables (social, medical, and mental health), homelessness was no longer significantly associated with either moderate or high ED use at *P* < 0.05. A further analysis in which past year homelessness was forced into the model to assess the point estimate of the effect size association of recent homelessness with ED use showed an AOR for moderate ED use of 1.13 (95% CI 0.91–1.40, not significant) and AOR 1.27 (95% CI 0.79–2.03, not significant) for high ED use (Figure 1).

Closer examination of the final model (Table 4) showed a notable commonality in variables associated with both moderate and high ED use (Table 4). Variables with the highest associations with *moderate* use included number of injuries (AOR 1.79, 95% CI 1.73–1.86, standardized regression coefficient [SRC] = 0.8), number or medical conditions (AOR 1.33, 95% CI 1.30–1.37, SRC = 0.18), and Medicaid insurance (AOR 1.49, 95% CI 1.37–1.62, SRC = 0.07).

**Table 4.** Stepwise multinomial logistic regression models of the association of ED use and social, medical, psychiatric, and substance use disorders.

Variable	Moderate ED use vs Non-use^*^		Variable	High ED use vs Non-use^*^	
	OR (95% CI)	Standardized regression coefficient		OR (95% CI)	Standardized regression coefficient
Number of injuries	1.79 (1.73–1.86)	0.8	Number of injuries	1.82 (1.75–1.89)	0.82
Medical conditions	1.33 (1.30–1.37)	0.18	Short Form-12 physical component	0.95 (0.94–0.96)	−0.32
Short Form-12 physical component	0.98 (0.977–0.983)	−0.12	Medical Conditions	1.53 (1.43–1.64)	0.27
Medicaid insurance	1.49 (1.37–1.62)	0.07	College education	0.47 (0.32–0.68)	−0.19
Short Form-12 mental component	0.99 (0.987–0.993)	−0.06	Married or cohabitating	0.56 (0.45–0.69)	−0.16
Black	1.34 (1.23–1.46)	0.05	Short Form-12 mental component	0.98 (0.966–0.984)	−0.14
Any traumatic experience	1.2 (1.13–1.27)	0.05	Medicaid insurance	1.97 (1.55–2.51)	0.11
College education	0.81 (0.76–.87)	−0.05	Parent with drug use history	1.95 (1.40–2.71)	0.09
Past year suicide attempt	3.03 (1.74–5.27)	0.03	Black	1.56 (1.19–2.05)	0.08
VA Tricare	1.29 (1.14–1.46)	0.03	Racial discrimination in the past year	1.35 (1.14–1.59)	0.07
Parent with prison history	1.23 (1.11–1.37)	0.03	Police trouble in past year	2.06 (1.25–3.38)	0.05
Borderline personality disorder	1.20 (1.09–1.21)	0.03	Past year homelessness	^**^	^**^
Social support	1.14 (1.07–1.21)	0.03	Past year suicide attempt	^**^	^**^
History of child sexual abuse	1.03 (1.01–1.04)	0.03	VA Tricare	^**^	^**^
Past year greater than one substance use disorder diagnosis	1.28 (1.08–1.52)	0.02	Past year greater than one substance use disorder diagnosis	^**^	^**^
Married or cohabitating	0.91 (0.86–0.97)	−0.02	Parent with prison history	^**^	^**^
Past year homelessness	^**^	^**^	Any traumatic experience	^**^	^**^
Police trouble in past year	^**^	^**^	Borderline personality disorder	^**^	^**^
Parent with drug use history	^**^	^**^	Social support	^**^	^**^
Racial discrimination in the past year	^**^	^**^	History of child sexual abuse	^**^	^**^

* All variables with a *P*-value for Wald chi-square <.01.

** Variable not included in the final stepwise regression.

*ED*, emergency department; *OR*, odds ratio; *CI*, confidence interval; *VA*, Veterans Administration.

Variables with the strongest independent associations with *high* ED use also included number of injuries in the past year (AOR = 1.82, 95% CI 1.75–1.89, SRC = 0.82), number of medical conditions (AOR = 1.53, 95% CI 1.43–1.64, SRC = 0.27), and Medicaid insurance (AOR = 1.97, 95% CI 1.55–2.51, SRC = 0.11), along with parental drug use history (AOR 1.95, 95% CI 1.40–2.71; SRC = 0.09). The strongest protective variables for both moderate and high ED use included high SF-12 component scores, college education, and being married or cohabitating.

Comparison of −2 log likelihood indicators of model fit showed the model of homelessness alone (−2LL = −41495, degrees of freedom [df] = 2) had a poorer goodness of fit than both the model of social (−2LL = 38,777, df = 40) and the model of medical and mental health factors alone (−2LL = 36,076, df = 52), and all three had a poorer model fit than the final combined stepwise model (−2LL = 35,188, df = 42) with each model fit significantly superior to that of the previous model at *P* < 0.005.

## DISCUSSION

This study showed that homelessness was strongly associated with ED use in an unadjusted model, as has been found in many other studies.^9^^–^^14^ However, estimates of the *independent* association of homelessness and ED use, adjusting first for measures of demographic characteristics and social disadvantage and then separately for medical and mental health, showed that both sets of factors largely accounted for this association. This suggests an important potential mediating role of these factors. The association of homelessness with ED use was further reduced to non-significance when both types of factors were included as covariates.

The strongest risk factors in the final model were injuries, medical conditions, Medicaid coverage, and parental drug use while the strongest protective variables were high physical- and mental health-related quality of life, college education, and being married or cohabitating. These findings are consistent with existing literature that has demonstrated lower socioeconomic status, lower educational attainment, public insurance, and poorer perceived health were predictors of frequent ED use.^29^ Physical injuries have also been shown to be associated with frequent ED visits, including return visits.^30^

The strong unadjusted association between homelessness and ED use is consistent with prior literature.^15^^,^^31^ However, in this study we further considered medical and social factors as separate blocks to explore the association of homelessness and ED use adjusting for these factors. Additionally, our study was based on a nationally representative sample extending its generalizability to populations that included people outside clinical settings.^17^^,^^18^ The NESARC-III dataset was also exceptional in the rich array of social variables unavailable in medical records (eg, education, parental histories, adverse childhood events, social isolation, and criminal justice interaction.)

### Implications

It has been suggested that the high cost of healthcare in the US compared to other wealthy countries reflects limited provision of social services.^5^ Health policy experts increasingly recognize the social determinants of health, and federal and local initiatives are emerging to address social needs and reduce healthcare service use and costs, including ED costs.^32^^,^^33^ While frequent ED users represent only 4–8% of ED patients, they account for 21–28% of all ED visits and generate significant costs.^34^ Recent studies show that individualized case management interventions can modestly reduce ED use.^35^^–^^37^ Other studies that focus on primary care access are less promising since most frequent ED users already use high levels of primary care.^15^ Housing-focused initiatives significantly reduce homelessness but have had limited effect on the physical or mental health of clients, on decreasing ED use, or on reducing health service costs. ^38^^–^^42^ These mixed findings suggest there is a larger context beyond service integration and supported housing that requires attention.

The concurrence of homelessness, social disadvantage, and chronic medical and mental illness points to a vulnerability deeper than merely having multiple, chronic illnesses and may be best understood through the evolving concept of allostatic burden.^43^ Allostasis is the general adaptive capacity of a person to respond effectively to physical or social demands. Allostatic burden refers to the magnitude of the demand for and potential failure of adaptive capabilities. In individuals with high allostatic burden, the cumulative effect of chronic stress and life events overwhelms adaptive capacities in a broad sense. Allostatic burden has been shown to be associated with poorer health outcomes in cardiovascular disease, diabetes, preeclampsia, geriatric frailty, periodontal disease, PTSD, psychotic disorders, and alcohol dependence,^43^ and to arise from conditions of poverty, segregation, discrimination, sexual trauma, and low educational attainment and thus exceeds any conception of chronic disease that merely reflects illnesses continuing over a long-term course.^43^ Many indicators of allostatic burden were significant in our model of high ED use and are disproportionately represented in the homeless population. While no studies to date have examined the association of allostatic load and frequent ED use, the allostatic burden model may facilitate understanding of frequent ED use, and specifically high use among people experiencing homelessness.

In recognition of what is currently known, social emergency medicine (EM) should be added to the EM research agenda and included in the core curriculum for ED residents via both didactics and community-based learning.^44^ A useful framework could differentiate three distinct levels of care: acute care for immediate problems (eg, appendicitis, traumatic injuries); acute-on-chronic care for urgent treatment of exacerbated heart failure; diabetic ketoacidosis, etc; and care for long-term overwhelming allostatic burden, the complex of lifelong social and medical problems that challenges the ability of an individual to maintain themselves in the society in which they live, and about which much remains to be learned.^2^^,^^44^

## LIMITATIONS

Several limitations warrant consideration. First, specific data on the immediate reasons for individual ED visits were not available in NESARC-III. While medical and mental health problems account for much of the association between ED use and homelessness, it is unclear whether ED visits were directly related to treatment of these health issues. Previous studies found that the majority of visits among patients with mental illness were for physical health conditions rather than reasons related to mental health.^45^^–^^48^ We heuristically separated medical and mental health problems but recognize that they are tightly intertwined.^49^^–^^51^

Second, our study was cross-sectional and cannot support conclusions about causality. The variable representing homelessness referred to prior year homelessness without data on the recency or chronicity of the homelessness episode. Additionally, our cross-sectional data is from 2012–2013 and associations may have changed in the intervening time. Our findings suggest trends to be explored in longitudinal studies of how ED use among homeless adults, as well as others, relates to overwhelming long-term allostatic burden.

Third, the sample excluded institutionalized adults, omitting pertinent populations at high risk for homelessness such as incarcerated individuals and those in homeless shelters. This limitation is not unique to our study, although it is more comprehensive than in previous literature. Finally, some NESARC-III variables themselves are imprecise and of uncertain validity. Homelessness and ED use were based on self-report and thus subject to recall bias. The ED use item was limited to a maximum of 10 or more visits per year, limiting the precision with which we could analyze the construct of “high” ED use. It is possible that at the extremes of ED use, there may have been an even stronger association with homelessness and other evidence of extreme allostatic burden.^15^

## CONCLUSION

Homeless individuals use the ED at higher rates than other individuals, but when adjusting for other social and medical factors, we did not find an independent association between homelessness and higher ED usage. This highlights the complex interplay between social and medical issues and should encourage the development and evaluation of more fully integrated training and service delivery models linking these intersecting dimensions of need.
